# Effect of COVID-19 on Retinal and Choroidal Microvasculature in the Pediatric Population: A Literature Review

**DOI:** 10.3390/medicina62081603

**Published:** 2026-08-21

**Authors:** Evita Evangelia Christou, Jane L. Ashworth, Noha M. Soliman, Peter Kiraly, Tariq Aslam

**Affiliations:** 1 Department of Ophthalmology, Manchester Royal Eye Hospital, Manchester University NHS Foundation Trust, Manchester M13 9WL, UK; 2 Department of Ophthalmology, Oxford Eye Hospital, Oxford University Hospitals NHS Foundation Trust, Oxford OX3 9DU, UK; 3 School of Health Sciences, University of Manchester, Oxford Road, Manchester M13 9PL, UK; 4 Institute of Ophthalmology, University College London, London WC1E 6BT, UK; 5 NIHR Biomedical Research Center, Moorfields Eye Hospital and UCL Institute of Ophthalmology, London EC1V 2PD, UK

**Keywords:** COVID-19, SARS-CoV-2, multisystem inflammatory syndrome children, MIS-C, pediatric, optical coherence tomography angiography, retinal and choroidal microvasculature, choriocapillaris, macula capillary plexus, peripapillary capillary plexus, vessel density, foveal avascular zone

## Abstract

*Backgroundand Objectives*: Retinal microcirculation may serve as a surrogate marker of systemic vascular status and can be non-invasively assessed using optical coherence tomography angiography (OCTA). Emerging evidence suggests that retinal and choroidal microvascular alterations may occur following coronavirus disease 2019 (COVID-19) and multisystem inflammatory syndrome in children (MIS-C), potentially reflecting systemic vascular and inflammatory processes. This review summarizes the current evidence regarding retinal and choroidal microvascular changes detected by OCTA in pediatric patients with COVID-19 and MIS-C. *Materials and Methods*: A structured literature search of the National Center for Biotechnology Information (NCBI) PubMed database was conducted from inception until April 2026. Original English-language studies evaluating retinal and choroidal microcirculation using OCTA in pediatric patients with COVID-19 or MIS-C were identified and reviewed. *Results*: Available studies suggest variable retinal and choroidal microvascular alterations following COVID-19 and MIS-C, including changes in vessel density, perfusion, and foveal avascular zone parameters. Several investigations reported reduced vessel density, particularly within the deep capillary plexus, whereas others demonstrated increased peripapillary vascular parameters or no significant differences during the observation period. Differences in patient characteristics, disease severity, timing of imaging, OCTA protocols, and study design likely contribute to the heterogeneity of the reported findings. *Conclusions*: OCTA represents a promising research tool for investigating retinal and choroidal microvascular alterations associated with pediatric COVID-19 and MIS-C. However, the available evidence remains limited by small observational studies, methodological heterogeneity and inconsistent findings. Larger prospective longitudinal studies using standardized imaging protocols are needed to determine the clinical significance and reproducibility of OCTA-derived parameters and to establish whether they may have potential as biomarkers of ocular or systemic vascular involvement.

## 1. Introduction

Coronavirus disease 2019 (COVID-19), caused by severe acute respiratory syndrome coronavirus 2 (SARS-CoV-2), is increasingly recognized as a multisystem disease affecting the respiratory, cardiovascular, neurological, renal, and ocular systems [1,2,3]. Endothelial dysfunction, inflammation, immune dysregulation, and coagulation abnormalities have been proposed as major contributors to systemic microvascular injury [4,5,6,7,8]. Although children generally experience milder acute infection than adults, a subset develops multisystem inflammatory syndrome in children (MIS-C), a post-infectious hyperinflammatory condition characterized by widespread vascular involvement and immune activation [9,10,11].

The retina provides a unique opportunity to evaluate systemic microvascular health because its circulation can be visualized non-invasively. Optical coherence tomography angiography (OCTA) enables high-resolution assessment of retinal and choroidal capillary networks and quantitative analysis of vessel density, perfusion, and foveal avascular zone (FAZ) characteristics. OCTA has therefore emerged as a valuable tool for investigating retinal microvascular alterations associated with systemic vascular diseases [12,13,14,15].

Pediatric studies have reported retinal and choroidal microvascular alterations following COVID-19 and MIS-C, although findings remain inconsistent regarding the direction of vascular changes. Variability in disease severity, timing of OCTA assessment, imaging protocols, patient age, and methodological approaches may all contribute to these discrepancies. Furthermore, the evidence is derived predominantly from small observational studies, limiting conclusions regarding the clinical significance of these findings [16,17,18,19,20,21,22,23,24,25,26,27,28].

This narrative review summarizes current evidence on retinal and choroidal microvascular alterations detected by OCTA in pediatric patients with COVID-19 and MIS-C, discusses potential pathophysiological mechanisms, evaluates the heterogeneity of the available evidence, and identifies directions for future research [16,17,18,19,20,21,22,23,24,25,26,27,28].

## 2. Methodology

A structured literature search was conducted in the PubMed database (National Center for Biotechnology Information, NCBI) from inception through April 2026 without restrictions on publication year. The search was limited to English-language publications. Relevant keywords were combined using the Boolean operators AND and OR. The search strategy included combinations of the following terms: (‘COVID-19’ OR ‘SARS-CoV-2’) AND (‘multisystem inflammatory syndrome children’ OR ‘MIS-C’) AND ‘pediatric’ AND (‘optical coherence tomography angiography’ OR ‘OCTA’) AND (‘retinal and choroidal microvasculature’ OR ‘macula capillary plexus’ OR ‘peripapillary capillary plexus’ OR ‘vessel density’ OR ‘foveal avascular zone’). The reference lists of eligible articles were also manually screened to identify additional relevant studies.

The search strategy was designed to identify studies evaluating retinal and choroidal microcirculation using OCTA in pediatric patients with confirmed COVID-19 or MIS-C. Studies were eligible if they met the following criteria: (1) original studies conducted in the pediatric population; (2) confirmed diagnosis of SARS-CoV-2 infection or multisystem inflammatory syndrome; (3) utilization of OCTA imaging. Studies were excluded if they: (1) had an uncertain or unconfirmed diagnosis; (2) were not published in English; (3) contained insufficient or incomplete data; or (4) included participants older than18 years. Duplicate publications from overlapping patient cohorts were excluded.

Titles, abstracts and subsequently full-text articles were independently screened by two reviewers (EEC, PK) according to the predefined eligibility criteria. Any disagreements regarding study eligibility were resolved by discussion and consensus.

Eligible studies were reviewed and data extracted from the selected articles were synthesized in the present review. To the best of our knowledge, this is the first review evaluating retinal and choroidal microcirculation assessed by OCTA in pediatric patients with COVID-19 and MIS-C.

**Figure 1:**
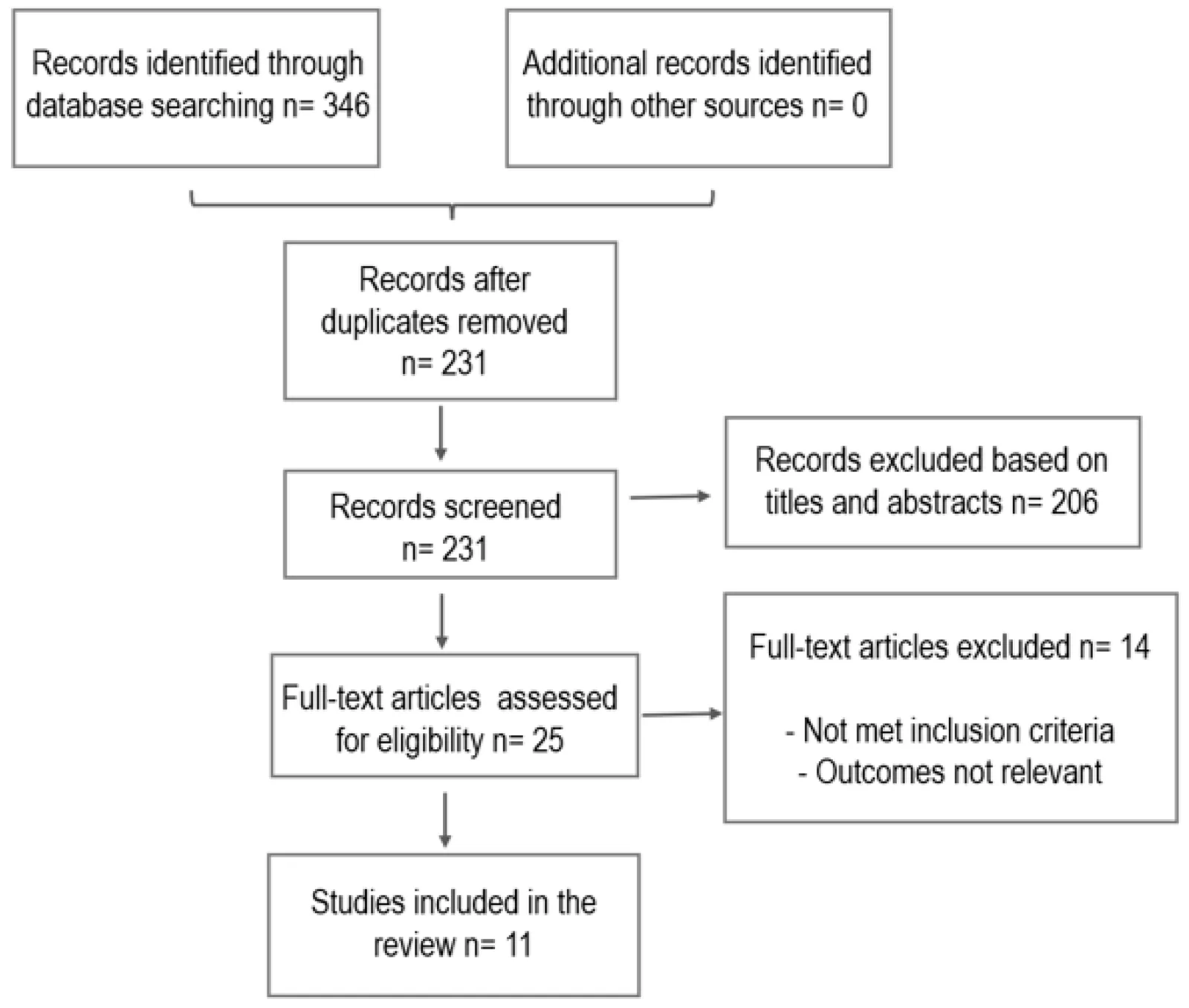
Study selection flow diagram.

This study represents a narrative review based on a structured literature search and was not designed as a formal systematic review. The study selection flow diagram (Figure 1) is included to enhance transparency of the literature identification and selection process and should not be interpreted as indicating formal adherence to systematic review methodology.

## 3. Results

### 3.1. Pathophysiological Mechanisms in COVID-19 and MIS-C

COVID-19 is a systemic disease characterized by direct viral invasion and immune-mediated inflammation. The underlying pathophysiological mechanisms mainly involve widespread endothelial inflammation, while microvascular dysfunction, immune dysregulation and coagulation abnormalities are considered key features of the disease and microvascular injury. These vascular alterations may also affect the retinal and choroidal circulation, making the ocular tissue a potential site for investigating microvascular involvement [29,30,31].

The angiotensin-converting enzyme 2 (ACE-2) receptor serves as the primary entry point for SARS-CoV-2 into host cells, facilitating viral infection. In humans, ACE-2 is widely expressed in multiple tissues, including the pulmonary, intestinal, renal, and endothelial cells. Given that COVID-19 is associated with a hypercoagulable state and an increased inflammatory response, the widespread expression of ACE-2 receptors, particularly within endothelial cells, appears to play a central role in disease pathogenesis. Endothelial cells are essential for maintaining vascular homeostasis through the regulation of vascular tone, coagulation, tissue perfusion and vascular permeability. Consequently, endothelial dysfunction induced by SARS-CoV-2 infection may lead to microvascular impairment, ischemic injury, tissue edema, and activation of a pro-inflammatory cytokine cascade, thereby contributing to the systemic manifestations of COVID-19 [32,33,34,35,36].

The ACE-2 receptor is also expressed in multiple ocular tissues, including the conjunctiva, cornea, ciliary body, retinal pigment epithelium (RPE), choroid and retina, and plays an essential role in retinal neurovascular function. SARS-CoV-2 exhibits a high affinity for ACE-2 expressed on Müller cells, RPE cells, and pericytes of retinal and choroidal endothelial cells. Viral infection together with the associated immune-mediated inflammatory response may exacerbate endothelial dysfunction and apoptosis, potentially promoting retinal and choroidal microthrombotic events. A previous immunohistochemical study indicated endothelial disruption and the presence of microthrombi in post-mortem samples, while the detection of viral RNA in retinal specimens has provided further evidence of viral presence in retinal tissue [32,33,34,35,36].

Children appear less susceptible to SARS-CoV-2 infection and generally exhibit milder disease compared with adults. This difference may be partially attributed to higher expression of ACE-2 receptors in children, which facilitates the degradation of angiotensin II. In contrast, lower baseline ACE-2 expression in adults, which may be further reduced following viral entry, may result in increased angiotensin II levels, thereby promoting vasoconstriction, inflammation, and fibrosis, and potentially contributing to greater disease severity [32,33,34,35,36].

A subset of pediatric patients developed rapidly evolving systemic inflammation which was initially reported in April 2020 [9,10,11]. Current evidence supports a model of post-infectious immune dysregulation triggered by prior exposure to SARS-CoV-2. A hyperinflammatory response, often referred to as ‘cytokine storm’, appears to play an important role in disease pathogenesis, with activation of the innate immune system initiating a cascade of inflammatory events and vascular involvement. In contrast to acute COVID-19, retinal microvascular alterations in MIS-C are thought to reflect immune-mediated endothelial injury rather than direct viral effects. Cytokine release, complement activation and systemic inflammation may contribute to transient retinal vascular dysfunction, although the precise mechanisms remain incompletely understood. Cytokine levels and immune cell abnormalities generally normalize during follow-up, suggesting a transient yet intense state of immune activation [9,10,11].

### 3.2. Effect of Hypoxia on Retinal and Choroidal Microvasculature

Hypoxia has been proposed as a mechanism contributing to retinal and choroidal microvascular alterations following COVID-19 and MIS-C. Systemic hypoxemia along with endothelial dysfunction, inflammation, immune dysregulation and coagulation abnormalities may affect retinal vascular homeostasis and tissue perfusion. These abnormalities are therefore likely multifactorial, reflecting the combined effects of SARS-CoV-2 and hypoxic stress rather than a single pathogenic pathway [29,30,31].

The retina is one of the most metabolically active tissues and requires continuous oxygen delivery to maintain neuronal function. Retinal blood flow is regulated by autoregulatory mechanisms that preserve tissue perfusion despite physiological fluctuations in systemic oxygenation and blood pressure. Nevertheless, severe systemic disturbances may impair autoregulation, resulting in altered retinal and choroidal blood flow [29,30,31].

Several biological pathways may contribute to these changes. Endothelial dysfunction may reduce nitric oxide bioavailability and promote vasoconstriction, while inflammatory cytokines can increase vascular permeability and disrupt the blood–retinal barrier. Hypercoagulability and platelet activation may contribute to microvascular obstruction, whereas oxidative stress and sympathetic activation may further compromise retinal perfusion. These mechanisms may influence OCTA-derived vascular parameters, although direct evidence linking individual mechanisms with specific OCTA findings in pediatric patients remains limited [15,29,30,31].

**Figure 2:**
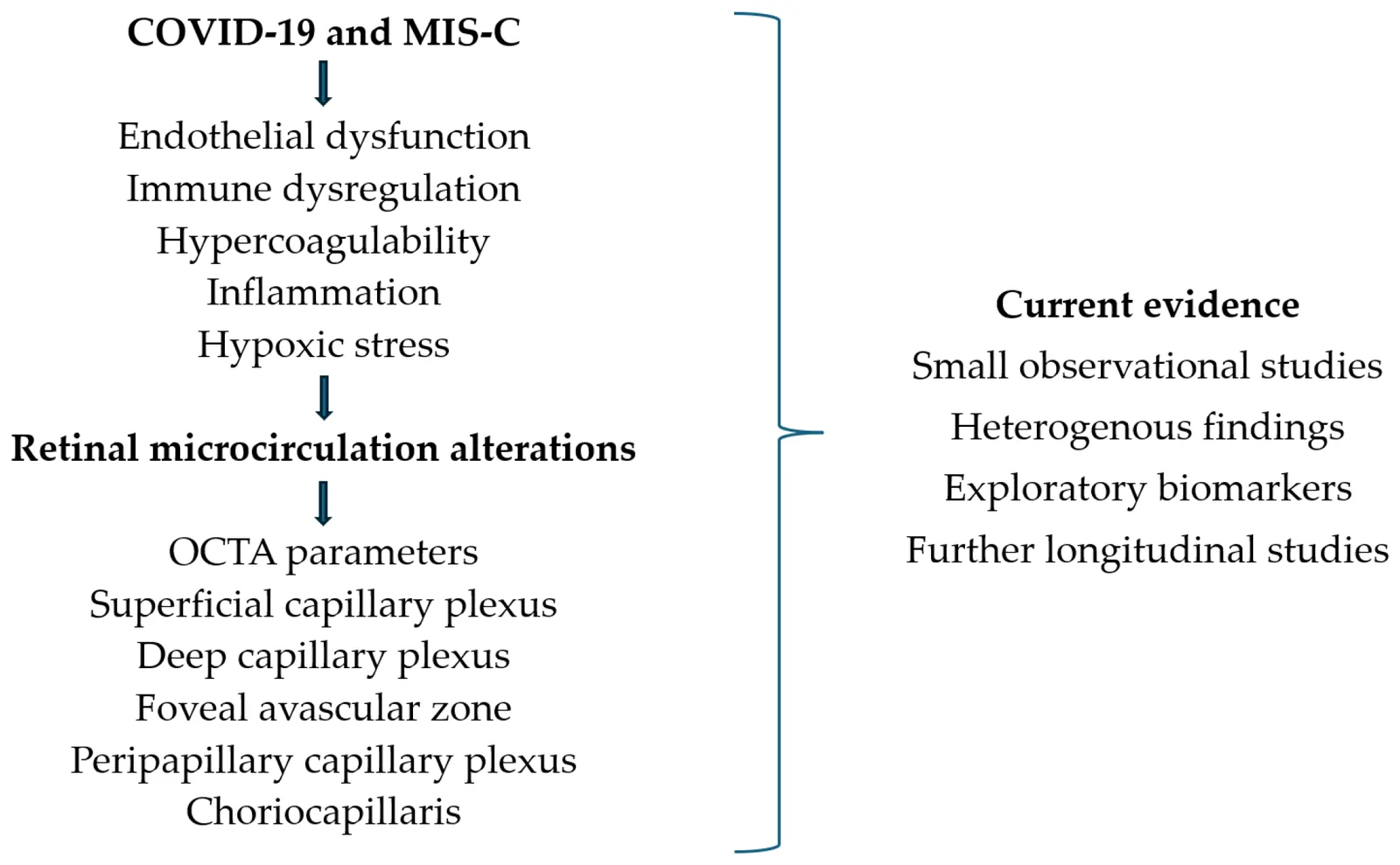
Proposed mechanisms and OCTA findings.

Retinal vascular plexuses may respond differently to systemic vascular stress. The deep capillary plexus (DCP) appears more susceptible than the superficial capillary plexus (SCP), possibly because of its anatomical location, higher metabolic demands, and finer vascular architecture. Alterations in FAZ parameters and regional differences between macular and peripapillary circulation have also been described, although findings remain inconsistent. Therefore, OCTA abnormalities should be interpreted as indicators of altered retinal perfusion rather than definitive evidence of ischemic injury or permanent vascular loss [29,30,31] (Figure 2).

Overall, hypoxic stress may contribute to retinal and choroidal microvascular alterations following COVID-19 and MIS-C, likely in combination with inflammation, endothelial dysfunction, immune dysregulation, coagulation abnormalities, and impaired vascular autoregulation [29,30,31].

### 3.3. Retinal Manifestations in SAR-CoV-2 Infection

Retinal manifestations associated with SARS-CoV-2 infection have been increasingly reported in the adult population, while evidence in pediatric patients remains limited [37,38,39,40,41,42,43,44,45,46,47,48,49,50,51,52]. Fundoscopic and structural imaging studies have described retinal hemorrhages, cotton wool spots, retinal vascular tortuosity, retinal vasculitis and vascular occlusions, optic neuritis and choroidal neovascularization. Pediatric evidence remains limited and is largely based on isolated case reports and small case series, precluding definitive conclusions regarding prevalence and causality [53,54,55,56,57].

Similarly, ocular involvement has been described in MIS-C, including retinal hemorrhages, retinal vascular tortuosity, vitritis and optic nerve involvement. Most reported abnormalities resolved following treatment, suggesting that they may represent transient inflammatory vascular alterations rather than persistent retinal findings [58].

### 3.4. Optical Coherence Tomography Angiography

OCTA is a non-invasive imaging modality that enables high-resolution visualization of the retinal and choroidal microvasculature without the need for intravenous dye injection. By detecting motion contrast generated by erythrocyte flow, OCTA provides quantitative assessment of the retinal and choroidal capillary plexuses, allowing evaluation of macular and peripapillary vascular networks. Consequently, OCTA has emerged as a valuable research tool for investigating retinal microvascular alterations associated with systemic vascular diseases, including COVID-19 and MIS-C [14,15].

Although OCTA is a valuable tool for detailed assessment of the retinal and choroidal microcirculation, certain inherent limitations should be considered when interpreting findings. OCTA measures flow-related signals rather than vascular anatomy directly; therefore, apparent reductions in vessel density may reflect altered perfusion, vasoconstriction, segmentation errors, motion artifacts or technical variability rather than true capillary loss. In addition, pediatric-specific factors including retinal vascular maturation, refractive status and axial length should be considered. A further limitation is the inability to evaluate patients with active SARS-CoV-2 infection or MIS-C due to the risk of infection transmission, thereby limiting the ability to capture dynamic vascular changes during the acute phase of the disease. Moreover, patient populations with variable severity of the disease may require face masks or have reduced physical activity (especially in acutely ill children) that may confound outcomes. Prolonged mask use is likely associated with hypercapnia that could potentially affect retinal and choroidal circulation. Finally, the technique requires full patient cooperation to obtain reliable results which may be particularly challenging in the pediatric population. Notwithstanding these limitations, OCTA remains a promising tool for assessing microcirculatory changes, which may reflect the underlying causes of ocular manifestations and serve as potential biomarkers for evaluating systemic vascular dysfunction [14,15,59].

### 3.5. Retinal and Choroidal Microvascular Alterations in Pediatric COVID-19

Several OCTA studies have evaluated retinal and choroidal microvascular changes in children following SARS-CoV-2 infection [16,17,18,19,20,21]. Although the reported findings are heterogeneous, most investigations suggest that retinal microcirculation may be altered during the early recovery period after COVID-19. However, the available evidence is derived almost exclusively from small observational studies, predominantly including children with mild disease, thereby limiting the generalizability of the findings.

The DCP appears to be the retinal vascular layer most consistently affected. Several studies reported reduced parafoveal DCP vessel density following COVID-19 infection, whereas changes in the SCP were less pronounced or absent [16,17,18,19,20]. The greater susceptibility of the DCP has been attributed to its distinct anatomical organization, higher metabolic demands, and increased vulnerability to alterations in retinal perfusion. Nevertheless, not all studies demonstrated significant DCP involvement, suggesting that these alterations are not universally present.

Macular vascular density and perfusion measurements have shown considerable variability. While several investigators observed reduced vessel density and perfusion within the macular region, others reported increased vascular parameters or no significant differences compared with healthy controls. Likewise, FAZ measurements demonstrated inconsistent findings, ranging from mild enlargement to no measurable differences. Given the substantial physiological variability of FAZ parameters, particularly in pediatric populations, these findings should be interpreted cautiously and should not be considered direct evidence of retinal ischemia [17,18,19,20,21].

Evaluation of the peripapillary circulation has produced similarly heterogeneous findings. Some studies demonstrated increased radial peripapillary capillary vessel density, whereas others reported unchanged or reduced values [18,19,21]. These discrepancies likely reflect differences in disease severity, timing of OCTA acquisition, vascular autoregulatory responses, imaging protocols, segmentation algorithms, OCTA platforms, and sample characteristics rather than true biological differences between patient populations.

Several studies also assessed structural OCT parameters and reported alterations in retinal nerve fiber layer thickness, ganglion cell layer thickness, and choroidal thickness. However, these findings remain inconsistent across studies and should be interpreted as indirect evidence of retinal and choroidal involvement rather than confirmation of permanent structural injury [17,19,60,61]. Similarly, changes in choroidal thickness represent an indirect marker of choroidal involvement and do not directly quantify choroidal microcirculation [20,21].

Overall, the available evidence suggests that retinal microvascular alterations may occur following pediatric COVID-19; however, the magnitude, direction, and persistence of these changes remain uncertain. Variability in patient age, disease severity, hospitalization status, timing of OCTA examination, OCTA devices, scan protocols, segmentation strategies, and relatively small sample sizes likely contribute to the heterogeneity observed among studies. Consequently, current OCTA findings should be regarded as exploratory observations rather than established biomarkers of disease severity or long-term vascular dysfunction.

### 3.6. Retinal and Choroidal Microvascular Alterations in MIS-C

The evidence regarding retinal and choroidal microvascular alterations in children with multisystem inflammatory syndrome in children (MIS-C) remains considerably more limited than that available for pediatric COVID-19 [22,23,24,25,50]. Most published studies are small prospective or cross-sectional investigations performed during the acute phase of the disease or within the first few months after recovery. Despite methodological differences, these studies consistently suggest that systemic hyperinflammation associated with MIS-C may be accompanied by transient retinal microvascular alterations detectable by OCTA.

Several studies reported reduced vessel density within the macular circulation, particularly involving the superficial and deep capillary plexuses, accompanied by reductions in retinal perfusion parameters. Nevertheless, the degree and distribution of vascular involvement differed considerably among studies. While some investigators demonstrated more pronounced alterations in the superficial capillary plexus, others observed greater involvement of the DCP or found no statistically significant differences compared with healthy controls [22,23,24,25,50]. These inconsistencies likely reflect differences in disease severity, timing of OCTA examination, imaging protocols, and study populations.

Changes in foveal avascular zone (FAZ) parameters have also been inconsistently reported. Mild enlargement of the FAZ has been described during the acute inflammatory phase in some cohorts, whereas other studies demonstrated no significant differences or normalization during follow-up. Considering the substantial physiological variability of FAZ measurements, these findings should be interpreted cautiously and should not be regarded as definitive evidence of retinal ischemia [22,23,24,25,50].

Evaluation of the peripapillary circulation generally demonstrated fewer abnormalities than those observed within the macular region. Most studies reported stable peripapillary vessel density throughout follow-up, although isolated investigations identified localized reductions or transient changes. Likewise, alterations in choroidal thickness varied considerably among studies and likely represent indirect manifestations of inflammatory or hemodynamic changes rather than direct evidence of impaired choroidal microcirculation [22,23,25].

Longitudinal observations suggest that several OCTA abnormalities may improve following clinical recovery, supporting the possibility that at least some retinal vascular alterations are reversible. However, persistent abnormalities have also been described in selected cohorts several months after MIS-C, indicating that complete vascular recovery may not occur in all patients. Whether these changes have long-term functional or prognostic significance remains unknown [22,23,24,25].

Overall, the available evidence indicates that retinal and choroidal microvascular alterations may occur in children with MIS-C; however, the current literature remains limited by small sample sizes, observational study designs, and methodological heterogeneity. Consequently, OCTA findings should be considered exploratory and hypothesis-generating until confirmed by larger prospective longitudinal studies using standardized imaging protocols.

### 3.7. Assessment of OCTA Findings in Pediatric COVID-19 and MIS-C

Although COVID-19 and MIS-C represent distinct clinical entities, both appear to be associated with retinal microvascular alterations detectable by OCTA. In pediatric COVID-19, reported changes have primarily involved the DCP, whereas studies in MIS-C more frequently describe alterations affecting both superficial and deep retinal vascular layers during the acute inflammatory phase. Nevertheless, considerable overlap exists between the two conditions, and no OCTA pattern can currently be considered disease-specific [16,17,18,19,20,21,22,23,24,25].

The heterogeneity observed across studies is likely multifactorial. Important sources of variability include differences in patient age, disease severity, hospitalization status, interval between diagnosis and OCTA examination, imaging devices, scan dimensions, segmentation algorithms, and image analysis software. In addition, normal developmental changes in the pediatric retinal vasculature, physiological interindividual variability, and relatively small study populations may further influence OCTA measurements. These factors should be carefully considered when interpreting published findings and comparing results across studies [16,17,18,19,20,21,22,23,24,25].

Overall, current evidence supports the presence of variable retinal and choroidal microvascular alterations following both COVID-19 and MIS-C; however, the available data are insufficient to establish a consistent vascular phenotype or determine whether OCTA abnormalities predict disease severity, systemic vascular involvement, or long-term ocular outcomes. Future multicenter longitudinal studies employing standardized imaging protocols will be essential to clarify these issues. The studies included in this review are summarized in Table 1, Table 2 and Table 3.

## 4. Discussion

The present review summarizes = current evidence regarding retinal and choroidal microvascular alterations detected by OCTA in pediatric patients following COVID-19 and MIS-C. Available studies suggest that retinal microcirculation may be affected after SARS-CoV-2 infection; however, findings remain heterogeneous and should be interpreted cautiously. Most evidence derives from small observational studies with variable inclusion criteria, imaging protocols, and follow-up intervals, limiting direct comparisons.

Among OCTA-derived parameters, alterations involving the DCP are most frequently reported, although changes in the SCP, peripapillary circulation, and FAZ have also been described. However, these alterations are inconsistent, and several studies reported normal OCTA findings or complete normalization during follow-up. Consequently, no single OCTA parameter can currently be considered a reliable biomarker of pediatric COVID-19 or MIS-C.

Several factors may contribute to variability among studies, including patient age, disease severity, hospitalization status, timing of OCTA examination, image acquisition protocols, segmentation algorithms, scan dimensions, OCTA platforms, and small sample sizes. Physiological maturation of the retinal microvasculature during childhood and adolescence may also influence OCTA measurements and should be considered when interpreting pediatric studies.

Current evidence does not permit definitive conclusions regarding the mechanisms underlying reported OCTA abnormalities. Although endothelial dysfunction, immune-mediated injury, coagulation abnormalities, altered vascular autoregulation, inflammation, and hypoxic stress have all been proposed, available evidence is largely observational and does not establish causality. OCTA findings should therefore be interpreted as potential alterations in retinal perfusion rather than direct evidence of retinal ischemia or permanent microvascular damage.

The clinical significance of OCTA findings remains uncertain. Although OCTA provides detailed, non-invasive assessment of retinal microcirculation and is a promising research tool, evidence is insufficient to support its routine use for diagnosis, prognosis, or monitoring of pediatric patients recovering from COVID-19 or MIS-C. Larger multicenter longitudinal studies using standardized imaging protocols are needed to establish the reproducibility, diagnostic accuracy, and prognostic value of OCTA-derived biomarkers.

## 5. Limitations

This review has several limitations. First, it represents a narrative review based on a structured literature search and therefore does not include the formal methodology of a systematic review, such as risk-of-bias assessment or quantitative meta-analysis. Second, the available literature is limited by the predominance of small observational studies with heterogeneous patient populations, imaging protocols, and follow-up intervals. Finally, differences among OCTA devices and image analysis software may have influenced the reported vascular parameters, limiting direct comparison between studies.

## 6. Conclusions

Retinal and choroidal microvascular alterations have been reported in pediatric patients following COVID-19 and MIS-C, suggesting a possible association between SARS-CoV-2-related systemic vascular and inflammatory processes and changes in the ocular microcirculation. However, the available evidence remains limited by small observational studies, methodological heterogeneity, and inconsistent OCTA findings.

At present, OCTA should be regarded primarily as a research tool for investigating retinal and choroidal microvascular alterations in this setting, rather than an established clinical biomarker. Future prospective multicenter studies using standardized imaging protocols, appropriate control groups and longer follow-up are needed to confirm the reproducibility of these findings, clarify their clinical significance and determine whether OCTA-derived vascular parameters have prognostic or biomarker potential in children affected by COVID-19 and MIS-C.

Key background points

- Emerging evidence suggests that SARS-CoV-2 may lead to microcirculation alterations in the macular
- and the peripapillary capillary plexus in children.
- OCTA characteristics may represent potential biomarkers for the long-term effects of hypoxia on retinal and choroidal tissue in the context of COVID-19 and MIS-C.
- Currently available data are limited; therefore, further research is warranted to clarify the role of microvasculature alterations in the underlying pathophysiology of ocular manifestations associated with COVID-19 infection.

## Figures and Tables

**Table 1:** Characteristics of included studies.

Study	Design	Population	Mean Age (Years)	N	Disease Severity	Time from Diagnosis to OCTA	OCTA Device
Comba et al. [16]	Case–control	COVID-19	13.5	30	Mild	1 month	AngioVue (Optovue) Fremont CA USA
Zengin et al. [17]	Prospective, cross-sectional, case–control	COVID-19	12.4	19	Mild	After recovery	RS-3000 Advance (Nidek) Aichi Japan
Guemes-Villahoz et al. [18]	Cross-sectional, case–control	COVID-19	12.0	27	Mild	4–8 weeks	Zeiss Cirrus 5000 Dublin CA USA
Demir et al. [19]	Prospective, cross-sectional, observational	COVID-19	13.1	16	Mild	35 days	AngioVue (Optovue) Fremont CA USA
Cetinkaya et al. [20]	Cross-sectional, comparative	COVID-19	13.3	27	Mild	12 weeks	DRI OCTA (Topcon) Topcon Tokyo Japan
Soysal et al. [21]	Prospective	COVID-19	4.5	40	Mild	3–6 months	XR Avanti AngioVue Fremont CA USA
Tugan (Yilmaz) et al. [22]	Prospective, cross-sectional, observational	MIS-C	11.0	34	Hospitalized	37 days	XR Avanti AngioVue Fremont CA USA
Incekalan et al. [23]	Prospective, comparative	MIS-C	12.0	21	Hospitalized	Acute and 60 days	XR Avanti AngioVue Fremont CA USA
Hizli et al. [24]	Prospective, case–control	MIS-C	11.9	60	Severe	Acute	XR Avanti AngioVue Fremont CA USA
Icoz et al. [25]	Cross-sectional, observational	COVID-19/MIS-C	9.0	41	Mixed	6 months	XR Avanti AngioVue Fremont CA USA
Kivrak et al. [50]	Retrospective, case–control	MIS-C	9.7	22	ICU	12 months	DRI Triton Plus Topcon Tokyo Japan

**Table 2:** Summary of OCTA findings.

Study	SCP	DCP	FAZ	Peripapillary Circulation	Choriocapillaris	Main Conclusion
Comba et al. [16]	No major change	↓	No significant change	Not evaluated	Not evaluated	Predominant DCP involvement
Zengin et al. [17]	↓	↓	Enlarged	Not evaluated	Indirect changes	Reduced macular perfusion
Guemes-Villahoz et al. [18]	↑	↑	Increased circularity	↑	Not evaluated	Increased vascular parameters
Demir et al. [19]	↓	↓	No change	↑	↓ flow	Macular parameters reduction with increased RPC
Cetinkaya et al. [20]	Slight ↓	Slight ↓	No change	Not evaluated	Slight ↓	Mild generalized reduction
Soysal et al. [21]	No change	↓	No change	↑	No change	Selective DCP involvement
Tugan et al. [22]	↓	Mild ↓	No change	No change	↓ flow	Acute MIS-C microvascular impairment
Incekalan et al. [23]	Mild ↓	↓	Enlarged	Mostly unchanged	Indirect changes	Improvement after recovery
Hizli et al. [24]	No change	↓	No change	Not evaluated	No change	DCP predominantly affected
Icoz et al. [25]	No significant differences	No significant differences	No change	No change	No change	Normalization by 6 months
Kivrak et al. [50]	↓	↓	No change	Not evaluated	Not evaluated	Persistent long-term reduction

**Table 3:** Overall synthesis and evidence.

OCTA Parameter	COVID-19	MIS-C	Overall Evidence	Major Sources of Heterogeneity
SCP vessel density	Variable (↓/↔/↑)	Variable	Inconsistent	Timing of imaging, disease severity
DCP vessel density	Frequently decreased	Frequently decreased	Moderately consistent	Scan protocol, OCTA platform
Macular perfusion	Usually reduced	Usually reduced	Moderate evidence	Imaging methodology
FAZ	Variable	Variable	Weak evidence	Physiological variability, age
Peripapillary circulation	Mixed findings	Mostly unchanged	Inconsistent	Different segmentation methods
Choriocapillaris	Limited evidence	Limited evidence	Insufficient	Few available studies
Structural OCT	Variable RNFL and choroidal thickness	Variable	Inconsistent	Follow-up interval

## Data Availability

Data available upon request from the corresponding author.
